# Hepatocyte protection and stellate cell control by *Salvia miltiorrhiza* in liver fibrosis

**DOI:** 10.3389/fphar.2026.2006283

**Published:** 2026-10-26

**Authors:** Yifan Liu, Wei Li, Weiwei Wu, Zihao Xu, Deng Wu

**Affiliations:** 1 Hubei University of Chinese Medicine, Wuhan, China; 2 Hubei Province International Science and Technology Cooperation Base for Innovative Research and Development of Food and Drug Resources and Targeted Delivery, Wuhan, China; 3 Wuhan No.7 Hospital, Wuhan, China; 4 Zengdu District People’s Hospital of Suizhou City, Suizhou, China; 5 Hubei Key Laboratory of Theoretical and Applied Research of Liver and Kidney in Traditional Chinese Medicine, Hubei Provincial Hospital of Traditional Chinese Medicine, Institute of Liver Diseases, Wuhan, China; 6 Hubei Provincial Clinical Research Center of Traditional Chinese Medicine for Liver Diseases, Wuhan, China; 7 Hubei Shizhen Laboratory, Wuhan, China; 8 Affiliated Hospital of Hubei University of Chinese Medicine, Wuhan, China

**Keywords:** hepatic stellate cells, hepatocytes, liver fibrosis, pharmacokinetics, Salvia miltiorrhiza, salvianolic acid B, tanshinones, therapeutic window

## Abstract

*Salvia miltiorrhiza* Bunge provides chemically distinct candidates for hepatocyte protection and hepatic stellate cell (HSC) control. This critical narrative Mini Review examines whether these effects occur with one defined intervention at compatible exposure. HSC control is distinguished as elimination, sustained deactivation after withdrawal or short-term state suppression. Same-study observations include hepatocyte protection with HSC state suppression and salvianolic acid A-induced apoptosis in PDGF-stimulated HSCs with preserved primary-hepatocyte viability. These findings provide partial support for cellular selectivity, but do not establish injured-hepatocyte rescue and HSC control within a pharmacologically supported exposure interval. Opposing ferroptosis effects among tanshinones further preclude class-level mechanistic inference. Clinical findings concern specific preparations and endpoints, and do not resolve the paired cellular question. Priorities are chemical and batch characterization, authenticated human models, validated fate endpoints, state- or subset-selectivity that preserves HSC homeostatic functions, and measurement of achieved exposure and target engagement. A useful window should meet separate hepatocyte-benefit, safety and HSC-control criteria, with donor-level effect estimates and confidence intervals. Distinguishing intrinsic susceptibility from delivery-mediated enrichment is essential for translating these candidates into reproducible antifibrotic interventions.

## 1 Introduction

Liver fibrosis involves reciprocal interactions between injured hepatocytes and activated hepatic stellate cells (HSCs) (Lu et al., 2023). Lineage tracing identified HSCs as the source of 82%–96% of myofibroblasts in tested mouse models of toxic, cholestatic and fatty liver disease (Mederacke et al., 2013). Spontaneous fibrosis resolution involves HSC apoptosis, declining TIMP expression and increased matrix degradation (Iredale et al., 1998). However, some HSCs survive and acquire an inactive state that remains more susceptible to reactivation than the original quiescent state (Kisseleva et al., 2012). Protecting hepatocytes while controlling fibrogenic HSCs is therefore an attractive therapeutic principle, provided that control preserves the functions needed for liver homeostasis.

Earlier reviews described hepatoprotection and antifibrotic effects of medicinal plants and *Salvia miltiorrhiza* (Duval et al., 2014; Yang et al., 2022). The unresolved question is whether both benefits occur with one intervention at compatible exposure. Findings from different plant metabolites cannot establish a shared cellular mechanism, and reduced α-smooth muscle actin (α-SMA), collagen or proliferation does not demonstrate HSC elimination. Rather than catalogue pathways already covered in broader reviews (Lan et al., 2025; Yang et al., 2025), we examine intervention identity, cell-specific outcomes and achieved exposure.

We distinguish HSC elimination, durable deactivation after withdrawal, and short-term suppression of the activated state. We also distinguish intrinsic cellular susceptibility from selectivity produced by altered drug distribution. These distinctions determine whether an apparent dual-cell benefit supports a pharmacological mechanism, a delivery strategy or only complementary observations (Figure 1).

**FIGURE 1:**
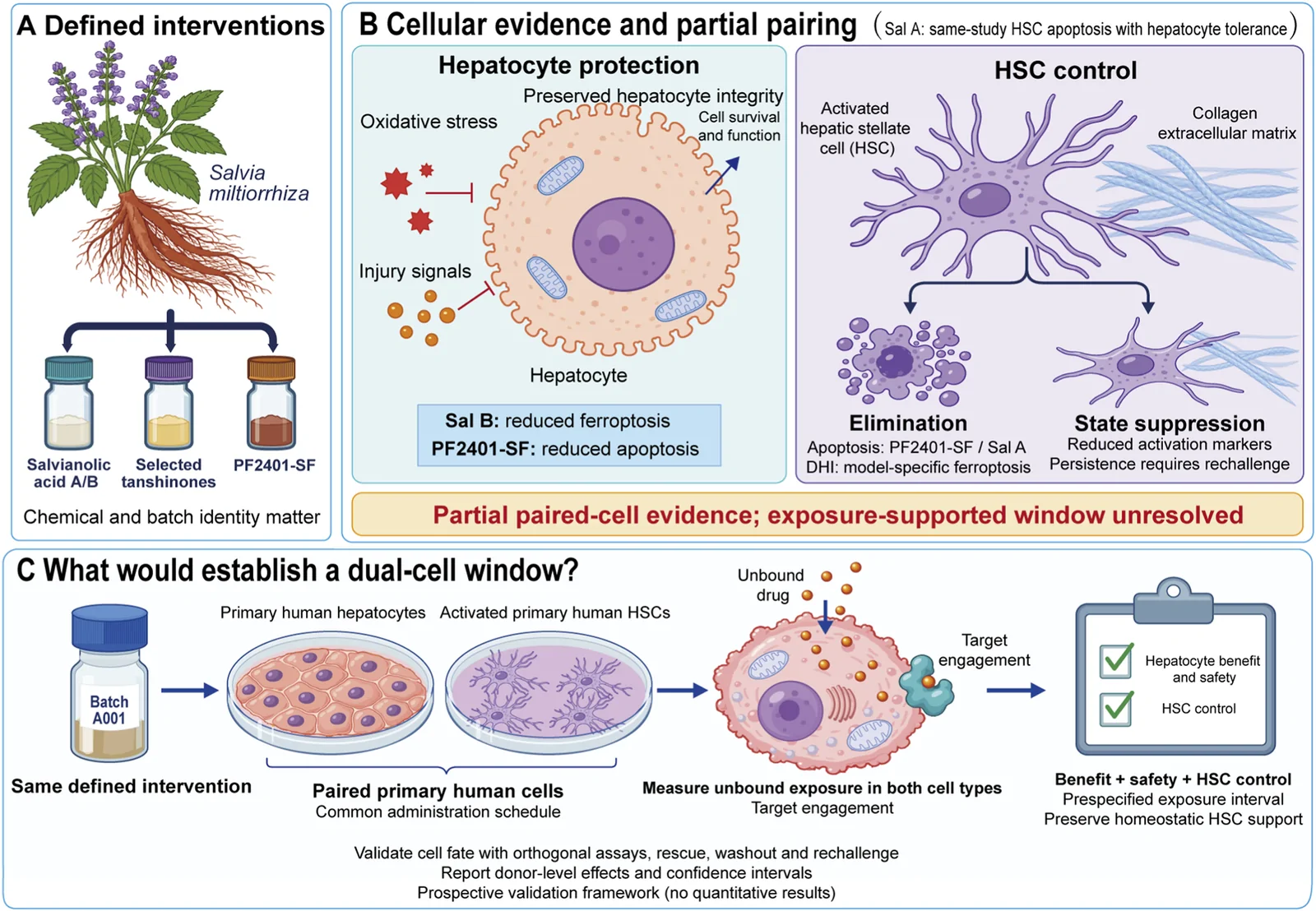
Evidence and validation requirements for dual-cell effects of *Salvia miltiorrhiza* in liver fibrosis. **(A)** Plant metabolites and fractions require distinct chemical and batch characterization. **(B)** Existing evidence includes hepatocyte protection and HSC control across studies, and same-study observations of protection with state suppression or Sal A-associated HSC apoptosis with primary-hepatocyte tolerance. Tanshinone ferroptosis effects depend on the tested chemical entity and model. **(C)** Prospective validation pairs authenticated human hepatocyte and HSC systems under a common administration design, measures achieved exposure and appropriate target engagement, and applies prespecified hepatocyte-benefit, safety and HSC-control criteria. Complementary systems include multicellular hiPSC liver models, liver-on-chip and human fibrotic liver slices. Functional state or subset selectivity should preserve homeostatic HSC support. Washout and rechallenge test persistence; donor-level estimates and confidence intervals quantify uncertainty.

In established fibrosis, hepatocyte protection may reduce injury without removing matrix-producing cells. HSC elimination could also impair homeostatic functions, whereas apparent deactivation may remain reversible. The relevant comparison therefore includes hepatocyte benefit and safety, fibrogenic HSC control, and preservation of supportive HSC functions.

We last searched PubMed, Web of Science Core Collection and CAS SciFinder on 7 October 2026. Supplementary Methods S1 and Supplementary Table S1 summarize data collection and selection. Additional source appraisal, clinical evidence, the ConPhyMP assessment index and prospective validation criteria are provided in Supplementary Tables S2, S4, S5 and S6, respectively. The individual ConPhyMP checklists are provided in Supplementary Data Sheet 1. English-language publications were selected during revision; the searches themselves had no added language filter. Yifan Liu and Wei Li used initial assessment by one author followed by itemwise checking by the other. Relevant reports examined Danshen-related hepatocyte injury, HSC responses or fibrosis, or informed exposure and delivery. This critical narrative review distinguishes partial paired-cell evidence from cross-study comparisons and does not claim exhaustive evidence coverage.

## 2 Botanical identity and pharmacological comparability

*Salvia miltiorrhiza* Bunge is an accepted species in Lamiaceae (Royal Botanic Gardens, Kew). Danshen, Salviae miltiorrhizae radix et rhizoma, comprises its dried root and rhizome (European Medicines Agency, 2022) and is traditionally used to promote blood circulation and resolve blood stasis (Lan et al., 2025). Contemporary forms include decoctions, injections, dripping pills and multibotanical formulas. These differ from enriched fractions and isolated plant metabolites in composition, route and exposure. Traditional use motivates investigation but does not establish preparation equivalence or clinical efficacy against liver fibrosis.

ConPhyMP guidance makes chemical characterization central to reproducibility (Heinrich et al., 2022). Evidence should identify the botanical source and medicinal part, extraction solvent, process and yield, quantitative chemical markers, and administration route, vehicle and formulation. Batch information and metabolite purity are also needed where applicable. Poorly characterized extracts may generate hypotheses, but cannot support metabolite-specific attribution or exposure comparisons.

Material continuity is especially important when comparing a traditional preparation with a standardized fraction or isolated metabolite. A formula or drug-containing serum cannot be attributed to Danshen without additional evidence separating its contribution. Conversely, activity of a purified metabolite does not validate the parent extract. Its amount, chemical context and availability in the preparation must support that attribution (Lan et al., 2025; Heinrich et al., 2022).

Different extraction and enrichment procedures change metabolite profiles. Hydrophilic salvianolic acids and lipophilic tanshinones differ in solubility, metabolism and exposure; a fraction’s activity cannot automatically be assigned to its major marker. Rat comparisons of pure tanshinones with a liposoluble extract found different plasma and tissue exposure despite comparable metabolite dosing (Wang et al., 2020). In fibrotic rats receiving FZHY, increased systemic Sal B exposure did not imply increased liver distribution (Yang et al., 2015). Material and disease-state continuity therefore matter to exposure comparisons.

PF2401-SF connects chemical characterization with hepatocyte protection, HSC apoptosis and rat pharmacokinetics across related studies (Parajuli et al., 2013; Park et al., 2008; Park et al., 2007; Park et al., 2009). The hepatocyte study reported 11.5% tanshinone I, 41.0% tanshinone IIA and 19.1% cryptotanshinone (Park et al., 2007). These proportions permit nominal metabolite-equivalent calculations for the reported material. Applying them to another study’s 20 μg/mL fraction is conditional on comparable composition, which is not established by the fraction name alone. Table 1 provides the compact equivalents; the calculation and provenance are retained in Supplementary Table S3.

**TABLE 1:** Available model and treatment conditions relevant to dual-cell activity. Concentrations are nominal unless stated otherwise. The PF2401-SF equivalents use the order tanshinone I/tanshinone IIA/cryptotanshinone and the composition reported by Park et al. (2007); the 20 μg/mL equivalents are conditional on comparable composition in the HSC study, not measurements of that experimental batch. No inferred quantity is presented as a measured exposure. Entries explicitly identify unresolved assay details. L02, LO-2 and HL-7702 observations do not establish authenticated human-hepatocyte safety Cellosaurus. L-02 (CVCL_6926). Reduced activation markers do not establish sustained deactivation.

Intervention	Hepatocyte model and conditions	HSC model and conditions	Comparability gap	Supported interpretation
PF2401-SF	Primary rat hepatocytes; 40 μg/mL (16.6/55.7/25.8 μM equivalents); 5-min pretreatment, GCDC 100 μM for 4 h (Park et al., 2007)	t-HSC/Cl-6; 20 μg/mL (conditionally 8.3/27.9/12.9 μM); 12 h, caspase-dependent apoptosis (Parajuli et al., 2013)	Different nominal doses and schedules; batch equivalence not independently established	Protection and HSC apoptosis across studies; paired exposure window not established
Sal B	Primary rat hepatocytes; CCl4 10 mM for 1 h; AST-release EC50 71.1 ± 7.2 μM (Lin et al., 2006b)	Primary rat HSCs with TGF-β1; 35–280 μM for 48 h; significant α-SMA/collagen reduction only at 280 μM (Lin et al., 2006b)	Separate direct-toxicity assay lasted 1 h, up to 104.5 μM	Same-study protection and state suppression; safety at 280 μM/48 h unresolved
Sal A	Primary rat hepatocytes; 0–20 μM, 48 h; no significant MTT viability decrease (Lin et al., 2006a)	PDGF-BB-stimulated HSC-T6; 10 μM, 24 h; TUNEL, sub-G0/G1 and caspase-related apoptosis (Lin et al., 2006a)	Same study and nominal concentration overlap; different medium and serum; no injury-rescue arm or paired exposure	HSC apoptosis with hepatocyte tolerance; partial stimulus dependence
Tanshinone IIA	L02 with TNF-α/ActD; 12.5 μM, 24 h; reduced apoptosis (Wu et al., 2019). Primary-rat-hepatocyte CCl4 protection in a separate study (Zhu et al., 2010)	LX-2 with TGF-β1; 25 μM, 24 h; no collagen I or α-SMA reduction despite p-ERK change (Wu et al., 2019)	Unequal concentrations; L02 identity limitation; primary-hepatocyte result is from another report	Protection does not establish concurrent HSC control
Cryptotanshinone	L02 viability affected at 40 μM (Hou et al., 2023); separate timing not verified. Primary-rat-hepatocyte lipid endpoints do not supply matched injury rescue (Yin et al., 2009)	LX-2; 2.5–20 μM, 24-h viability and apoptosis assays (Hou et al., 2023); 10 μM, 48-h activation/metabolic assays (Li et al., 2024).	L02 identity limitation; no paired injured-primary-hepatocyte arm or verified washout/rechallenge	HSC apoptosis and lipocyte-like changes; safety and persistence unresolved
DHI	No paired injured-hepatocyte rescue in the assessed HSC studies (Shen et al., 2025; Ge et al., 2017)	LX-2/primary rat HSCs; 10 μM, 6/12/24 h, state suppression (Ge et al., 2017). Primary mouse HSCs; 0.4/0.5 μM, 24-h viability with ferroptosis evidence (Shen et al., 2025)	Different species and endpoints; timing of every rescue panel not independently verified	State suppression and ferroptosis evidence; paired hepatocyte benefit remains untested
FZHY medicated serum	L02 with TNF-α/ActD; 10% serum, 24 h (Wu et al., 2019). Separate primary-hepatocyte DNA-transfer study (Tao et al., 2014)	LX-2 with TGF-β1; 10% serum, 24 h, reduced activation markers; no demonstrated increase in apoptosis (Wu et al., 2019)	Multibotanical serum mixture; L02 identity limitation; indirect injury-mediated routes (Tao et al., 2014)	Protection with state suppression; Danshen attribution and HSC autonomy unresolved

HSC, hepatic stellate cell; Sal A/B, salvianolic acid A/B; DHI, dihydrotanshinone I; FZHY, Fuzheng Huayu; GCDC, glycochenodeoxycholic acid.

## 3 Cellular evidence

### 3.1 Hepatocyte protection and HSC elimination

PF2401-SF reduced GCDC-induced apoptosis in primary rat hepatocytes (Park et al., 2007). Sal B reduced death-receptor-associated apoptosis in HL-7702 cells and injury in LPS/GalN-challenged mice (Yan et al., 2010). The cell experiment used 1 μM Sal B for 1 h before a 6-h ActD/TNF-α challenge. These acute protection findings do not establish paired HSC control. In bile duct-ligated rats, a Danshen water extract reduced hepatocyte apoptosis without changing α-SMA expression (Oh et al., 2002), showing that hepatocyte benefit need not coincide with a detectable HSC activation response.

Hepatocyte-specific *Ecm1 deletion* abolished Sal B’s antifibrotic effect in a CCl4 model, while *Ecm1 knockdown* abrogated ferroptosis protection in L02 cells (Fu et al., 2024). A separate metabolic dysfunction-associated steatohepatitis study used Sal B magnesium salt and supported SIRT1 pathway involvement and binding (Zhou et al., 2026). L02, LO-2 and HL-7702 observations require the identity qualification recorded by Cellosaurus. L-02 (CVCL_6926); they do not establish protection in authenticated human hepatocytes. This limitation does not invalidate independent primary-cell or animal evidence, but prevents using the cell lines as a reliable human-hepatocyte safety arm.

Separate studies support regulated death in activated-HSC models. PF2401-SF induced caspase-dependent apoptosis in t-HSC/Cl-6 cells at 20 μg/mL for 12 h. In a carbon-tetrachloride rat model, 50 mg/kg on days 13 and 14 reduced α-SMA staining and increased TUNEL-positive cells; these separate tissue readouts provide less direct cell attribution than the *in vitro* experiments (Parajuli et al., 2013). Tanshinone I induced mitochondrial apoptosis in an activated rat HSC line without a hepatocyte comparator (Kim et al., 2003). Cryptotanshinone induced endoplasmic-reticulum-stress-associated apoptosis in LX-2 cells, with pharmacological attenuation by salubrinal (Hou et al., 2023). DHI was associated with ALOX15-dependent ferroptosis, although its proposed direct interaction with EGR1 relied more heavily on computational evidence (Shen et al., 2025). Tanshinone IIA and IH764-3 studies also reported HSC apoptosis or cell-cycle arrest (Che et al., 2010; Liu et al., 2012; Pan and Wang, 2012; Zhang et al., 2002). Pharmacological or genetic rescue supports pathway involvement, whereas predicted binding requires independent confirmation.

Regulated cell-death direction cannot be inferred from the tanshinone class. Tanshinone I, tanshinone IIA and cryptotanshinone inhibited ferroptosis; mechanistic experiments implicated reduced tanshinones acting with NQO1 to detoxify lipid peroxyl radicals, with key target-identification and animal experiments using synthetic derivative 15–047 (Wang et al., 2023a). This directly opposes a class-wide pro-ferroptotic interpretation. Tanshinone IIA also protected primary rat hepatocytes against CCl4 injury (Zhu et al., 2010) and inhibited ferroptosis-associated injury in high-glucose podocytes (Zhu et al., 2024). Conversely, danshensu reduced HSC activation markers while increasing lipid peroxidation (Wang et al., 2023b), although discordant source reporting and absent death-rescue evidence preclude classifying this as verified HSC ferroptotic death. These findings show that chemical class alone does not predict hepatocyte protection or HSC elimination.

### 3.2 HSC state suppression and direct comparisons

HSC control frequently reflects changes in activation rather than elimination. Sal B reduced activation-related endpoints in studies involving MEF2, FGF19/FGFR4, non-coding RNA regulation, p300/CBP and PDGFRβ (Li et al., 2025; Liu et al., 2023; Tian et al., 2021; Wang et al., 2022; Zhang et al., 2019). Lipophilic fractions and salvianolic acid C provide further evidence for state suppression (Sun et al., 2026; Tang et al., 2022).

DHI disrupted YAP–TEAD signaling and stimulated autophagy-associated collagen processing, whereas another study linked Sal B-mediated suppression of activation to autophagy inhibition (Ge et al., 2017; Jiang et al., 2022). These differences require interpretation in their metabolite and cellular contexts (Hung et al., 2020; Liu et al., 2025). Cryptotanshinone also promoted a lipocyte-like phenotype through inhibition of STAT3/CPT1A-dependent fatty-acid oxidation (Li et al., 2024). Such phenotypes establish neither HSC death nor persistence of deactivation after treatment withdrawal. Metabolite identity, activation state, autophagic flux and the endpoint may explain the divergent autophagy findings; neither direction warrants a universal recommendation.

Same-study evidence is more informative than a comparison across unrelated reports, but its endpoint matters. Sal A at 10 μM for 24 h induced apoptosis in PDGF-BB-stimulated HSC-T6 cells, supported by TUNEL staining, sub-G0/G1 accumulation and caspase-related measurements (Lin et al., 2006a). In the same study, primary rat hepatocytes exposed to 0–20 μM for 48 h showed no significant decrease in MTT viability. Sal A alone did not induce apoptosis in unstimulated HSC-T6 cells, suggesting stimulus dependence. This comparison supports HSC apoptosis with hepatocyte tolerance; it did not test injured-hepatocyte rescue, authenticated quiescent HSCs or paired cellular exposure. Sal B was likewise tested in primary rat hepatocytes and HSCs within one report (Lin et al., 2006b), but the different concentration–time and endpoint conditions did not establish a common protection-and-control interval.

FZHY-medicated serum and selected metabolites were tested in TNF-α/actinomycin-D-injured L02 and TGF-β1-stimulated LX-2 cells (Wu et al., 2019). Tanshinone IIA reduced L02 apoptosis at 12.5 μM/24 h but changed p-ERK1/2 without reducing LX-2 collagen I or α-SMA at 25 μM/24 h. FZHY serum (10%) reduced activation markers without demonstrated LX-2 apoptosis. Neither unequal nominal metabolite doses nor multibotanical serum establishes a Danshen-specific exposure window. A related formulation extended cellular comparisons without isolating Danshen effects (Sun et al., 2022). L02 identity additionally limits interpretation as human-hepatocyte protection (Cellosaurus. L-02 (CVCL_6926)).

Cryptotanshinone reduced LX-2 viability across 2.5–20 μM over 24 h, whereas an effect on L02 viability was reported at 40 μM (Hou et al., 2023). This comparison did not test injured-hepatocyte rescue or matched intracellular exposure. The L02 identity limitation further prevents direct inference of safety or selectivity relative to authenticated human hepatocytes (Cellosaurus. L-02 (CVCL_6926)). In a 12-week thioacetamide protocol, PF2401-SF at 1 or 2.5 mg/kg orally, five days per week, preceded toxin administration by 2 h; the higher dose improved injury and fibrosis indices (Parajuli et al., 2015). These tissue-level outcomes support plausibility, but do not demonstrate regression of established fibrosis or selective HSC death with hepatocyte rescue. Table 1 aligns available model, concentration and timing information with each intervention’s evidential gap. Nominal medium concentrations are not measurements of intracellular unbound drug. Tissue improvement still leaves open whether HSC changes are direct or secondary to reduced hepatocyte injury.

## 4 Barriers to a therapeutic window

### 4.1 Intervention and endpoint validity

A dual-cell claim fails when otherwise favorable outcomes come from different interventions, disease states or schedules. Healthy cell-line tolerance cannot substitute for rescue of injured primary hepatocytes. Similarly, prevention or concurrent treatment cannot establish reversal of established fibrosis (Lee et al., 2006). Methodological limitations and publication-bias concerns in a preclinical tanshinone IIA meta-analysis reinforce the need to assess study quality rather than count positive findings (Ying et al., 2019).

Apoptosis and ferroptosis require concordant functional and morphological evidence, supported where feasible by pharmacological rescue or genetic perturbation. Reduced α-SMA or collagen indicates state suppression; sustained deactivation requires follow-up after washout and renewed fibrogenic stimulation. Cell-autonomous HSC action additionally requires direct response in an HSC preparation without injury-derived, macrophage or LSEC mediators. FZHY studies show indirect routes through DNA from protected hepatocytes and macrophage-conditioned medium (Tao et al., 2014; Ping et al., 2024). Conditioned-medium transfer with control of residual drug and cell-specific perturbation can separate these contributions from direct HSC pharmacology.

### 4.2 Cellular exposure and delivery

After oral PF2401-SF at 10 mg/kg in rats, total plasma tanshinone IIA and cryptotanshinone peaked at 2.78 and 0.66 ng/mL, approximately 9.45 and 2.23 nM, respectively (Park et al., 2008). Human pharmacokinetic studies likewise measure systemic exposure without resolving drug availability in either cellular compartment (Jia et al., 2010; Cheng et al., 2023).

Concentration also constrains mechanistic interpretation. Sal B inhibited ceramide glucosyltransferase (UGCG) with a reported IC50 of 159 μM and reduced HSC activation-related phenotypes (Li et al., 2023). This enzyme-inhibition concentration is not a threshold for matched hepatocyte protection and HSC control. Protein binding, intracellular availability and attainable exposure must be considered before assigning *in vivo* relevance. High nominal concentrations warrant scrutiny, although uptake and active metabolites may modify local exposure.

Plasma pharmacokinetics informs systemic plausibility, while whole-liver concentration mixes cell populations and total cellular uptake can include sequestered drug. Cellular accumulation combined with binding measurements can estimate intracellular unbound concentration (Mateus et al., 2013; Mateus et al., 2017). Recovery, extracellular carry-over, medium binding, cellular volume and parent-metabolite stability require validation in each system. Exposure relevant to an extracellular or cell-surface target differs from intracellular target exposure; neither a tissue concentration nor an engagement assay alone establishes a dual-cell window.

Delivery studies distinguish carrier targeting from drug exposure. Modified lipid nanocapsules increased systemic tanshinone IIA exposure and carrier-associated liver signals (Ashour et al., 2021). Globin nanoparticles increased LC–MS/MS-measured parent-drug plasma and whole-liver exposure versus matched-dose intravenous suspension and improved TAA-associated fibrosis (Meng et al., 2015). Whole-liver enrichment still does not resolve hepatocyte versus HSC exposure. A vitamin-A/cyclodextrin carrier increased fluorescent-probe uptake in LX-2 cells without measuring intracellular unbound DHI; its HL-7702 comparator shares the L02 identity limitation (Cellosaurus. L-02 (CVCL_6926); Niu et al., 2023).

Sal B-loaded albumin nanoparticles reduced proliferation and contraction in Ang-II-stimulated HSC cultures at 100 μM over 24 h and also increased apoptosis-associated readouts (Wang et al., 2026). A tanshinone IIA liposomal formulation improved fibrosis after four weeks of CCl4 modeling; its LO-2 cytotoxicity comparison remains subject to the same identity limitation (Cellosaurus. L-02 (CVCL_6926); Cai et al., 2025). These formulation-specific findings require comparison against free metabolite and measurement of exposure in both cellular compartments.

The assessed paired studies provide partial evidence for cellular selectivity but do not connect injured-hepatocyte rescue and HSC control to a common, pharmacologically supported exposure interval. Missing unbound-exposure or engagement measurements identify the next validation step; they do not erase observed tolerance or cell-fate effects.

## 5 Discussion

Healthy-volunteer studies provide preparation-specific exposure and tolerability information: intravenous depside salts were studied in 12 volunteers (Jia et al., 2010), and a placebo-controlled Sal B study reported no serious adverse events, although treatment-related events included liver-test abnormalities (Cheng et al., 2023). Patient surveillance of a polyphenol injection described as containing magnesium lithospermate B as its main active constituent identified abnormal liver function in 58 of 2,633 patients (2.2%), with Naranjo scores above 5 (Cheng et al., 2020). Comorbidity, co-medication and the absence of an unexposed comparator limit causal interpretation. These findings establish neither attributable excess risk nor antifibrotic efficacy or long-term safety in liver fibrosis.

Clinical efficacy is preparation- and endpoint-specific. Oral Sal B showed no significant histological advantage over interferon-γ in 60 evaluable chronic hepatitis B patients (Liu et al., 2002). A FZHY-plus-entecavir pilot favoured one-stage regression among 46 paired biopsies, but not median Ishak-stage reduction (Gui et al., 2020). In a chronic hepatitis C trial, the F3/F4 biopsy comparison was not significant (P = 0.24) (Hassanein et al., 2022). A *post hoc* analysis of two trials included 218 of 232 randomized patients with compensated HBV cirrhosis; adjunctive FZHY was associated with fewer composite liver events (adjusted HR 0.362, 95% CI 0.159–0.828) (Guo et al., 2026). Post-randomization exclusions and limited events constrain this result; it is an associated report, not two additional independent trials. These studies do not isolate Danshen’s contribution or connect patient outcomes to paired cellular exposure. The assessed English-language evidence does not establish chemically defined salvianolate/depside-salt antifibrotic efficacy.

A paired human experiment should use one characterized batch in injury-challenged primary hepatocytes and activated primary HSCs, with healthy hepatocytes and validated quiescent HSCs as state controls. Multicellular hiPSC-derived liver models, liver-on-chip systems and human fibrotic precision-cut liver slices are complementary prospective systems, provided that their cellular states and relevant endpoints are validated. A common administration schedule should be used while measuring the exposure actually achieved in each cell type; equal nominal treatment does not require identical intracellular concentrations.

The window should be prespecified as the exposure interval meeting separate minimum hepatocyte-benefit and HSC-control criteria while remaining below a defined hepatocyte-injury threshold. Donor-level concentration–effect estimates and confidence intervals should quantify the effects and uncertainty around overlap. Threshold sensitivity analysis, randomization, blinding and predefined exclusions improve interpretability. A ratio between unrelated viability assays or a high tolerated-toxicity threshold cannot define clinical safety.

HSC control should preserve functions needed for hepatocyte homeostasis. HSC depletion and HSC-specific *Rspo3 deletion* altered hepatocyte zonation, metabolism, liver size and regeneration (Sugimoto et al., 2025). Fibrogenic programmes should therefore be assessed alongside HSC viability, lipid-storage or quiescence-associated programmes (for example, LRAT, RBP1 and PPARG) and functional support of hepatocytes. RSPO3 expression alone cannot define a universally dispensable or protective subset. Washout and rechallenge should test persistence and reactivation susceptibility, because an inactive HSC can remain distinct from the original quiescent state (Kisseleva et al., 2012). Single-cell profiling is useful when mixed states obscure interpretation; a fate-transition claim requires lineage or equivalent longitudinal evidence.

For exposure and engagement, LC–MS/MS accumulation and cellular-binding methods can be adapted to the paired preparations (Mateus et al., 2013; Mateus et al., 2017). Hydrophilic Sal B requires attention to uptake, while lipophilic tanshinones require control of binding, adsorption and sequestration. CETSA and thermal proteome profiling can assess engagement without an engineered competitive tracer (Martinez Molina et al., 2013; Savitski et al., 2014); live-cell BRET requires a compatible tagged target and tracer (Robers et al., 2015). Negative thermal-shift results do not exclude binding, and thermal shifts require orthogonal interpretation. These are prospective method choices, not validated assays for every proposed metabolite or target. Comparing free and targeted formulations can then distinguish intrinsic susceptibility from distribution-mediated benefit.

Supplementary searches identified context-dependent exposure, partial paired-cell evidence and clinical endpoint limitations. English-language eligibility, targeted narrative selection and heterogeneous models limit coverage and inference.

*Salvia miltiorrhiza* provides distinct candidates for hepatocyte protection and HSC elimination or state suppression. Existing paired observations justify further study, but the next advance is to connect injured-hepatocyte benefit, fibrogenic HSC control and preserved homeostatic functions within one characterized intervention and measured exposure interval.

## Supplementary material

Supplementary Table 1 Supplementary Methods S1 and Tables S1–S6.

Supplementary Data Sheet 1 Official ConPhyMP Tables 1 and 2a for 19 original reports, indexed in Supplementary Table S5.
