# Supplementary material for: Hepatocyte protection and stellate cell control by Salvia miltiorrhiza in liver fibrosis
Source: Front Pharmacol. 2026 Oct 26;17:2006283. doi: 10.3389/fphar.2026.2006283 (PMC13746170; doi:10.3389/fphar.2026.2006283)
Supplement: Supplementary file 2 [file Table1.docx]

Supplementary Methods S1 and Tables S1 to S6

# S1. Data collection and selection

PubMed, Web of Science Core Collection and CAS SciFinder searches, including directed PubMed patient-preparation searches, were last updated on 7 October 2026. No language or article-type restriction was applied.

Yifan Liu and Wei Li performed screening, full-text acquisition and extraction using initial assessment by one author followed by itemwise checking by the other. This was verification rather than two independent duplicate screens. The agreed disagreement procedure uses source reinspection and discussion; unresolved disagreements may be adjudicated by a third author.

**S1.1. PubMed**

Core and update searches used Title/Abstract fields without language or article-type restrictions. Directed preparation searches included broader All Fields terms and literal Title/Abstract terms; broad mapping of salvianolate to salvianolic was not treated as product-specific identification.

**Q1. Core search**

("Salvia miltiorrhiza"[Title/Abstract] OR Danshen[Title/Abstract] OR salvianolic[Title/Abstract] OR tanshinone*[Title/Abstract] OR cryptotanshinone[Title/Abstract] OR dihydrotanshinone[Title/Abstract] OR "PF2401-SF"[Title/Abstract] OR "IH764-3"[Title/Abstract]) AND (hepatocyte*[Title/Abstract] OR "hepatic stellate"[Title/Abstract] OR "liver fibrosis"[Title/Abstract] OR "hepatic fibrosis"[Title/Abstract]) AND ("1900/01/01"[Date - Publication] : "2026/08/18"[Date - Publication])

**Q2. Update search**

("Salvia miltiorrhiza"[Title/Abstract] OR Danshen[Title/Abstract] OR salvianolic[Title/Abstract] OR tanshinone*[Title/Abstract] OR cryptotanshinone[Title/Abstract] OR dihydrotanshinone[Title/Abstract] OR "PF2401-SF"[Title/Abstract] OR "IH764-3"[Title/Abstract]) AND (hepatocyte*[Title/Abstract] OR "hepatic stellate"[Title/Abstract] OR "liver fibrosis"[Title/Abstract] OR "hepatic fibrosis"[Title/Abstract]) AND ("2026/08/19"[Date - Publication] : "2026/10/07"[Date - Publication])

**C01. defined preparation liver**

(salvianolate[All Fields] OR "salvianolic acid salt"[All Fields] OR "salvianolic acid salts"[All Fields] OR "salvianolic acids for injection"[All Fields] OR "salvianolic acid for injection"[All Fields] OR "depside salt"[All Fields] OR "depside salts"[All Fields] OR "Danshen polyphenolate"[All Fields] OR "magnesium lithospermate B"[All Fields]) AND ("Liver Diseases"[MeSH Terms] OR liver[All Fields] OR hepatic[All Fields] OR cirrhosis[All Fields] OR fibrosis[All Fields])

**C02. defined preparation without disease filter**

(salvianolate[All Fields] OR "salvianolic acid salt"[All Fields] OR "salvianolic acid salts"[All Fields] OR "salvianolic acids for injection"[All Fields] OR "salvianolic acid for injection"[All Fields] OR "depside salt"[All Fields] OR "depside salts"[All Fields] OR "Danshen polyphenolate"[All Fields] OR "magnesium lithospermate B"[All Fields])

**C03. Danshen injection liver patients**

(Danshen[All Fields] OR "Salvia miltiorrhiza"[All Fields] OR "Salvia injection"[All Fields]) AND (injection[All Fields] OR injectable[All Fields] OR intravenous[All Fields]) AND ("Liver Diseases"[MeSH Terms] OR liver[All Fields] OR hepatic[All Fields] OR cirrhosis[All Fields] OR fibrosis[All Fields]) AND ("Humans"[MeSH Terms] OR patient*[Title/Abstract] OR clinical[Title/Abstract] OR trial[Title/Abstract])

**C04. injection name without disease filter**

(salvianolate[All Fields] OR "salvianolic acids for injection"[All Fields] OR "salvianolic acid for injection"[All Fields] OR "depside salts"[All Fields] OR (("Salvia miltiorrhiza"[All Fields] OR Danshen[All Fields]) AND polyphenol*[All Fields] AND (inject*[All Fields] OR intravenous[All Fields])))

**C05. injection name liver**

(salvianolate[All Fields] OR "salvianolic acids for injection"[All Fields] OR "salvianolic acid for injection"[All Fields] OR "depside salts"[All Fields] OR (("Salvia miltiorrhiza"[All Fields] OR Danshen[All Fields]) AND polyphenol*[All Fields] AND (inject*[All Fields] OR intravenous[All Fields]))) AND ("Liver Diseases"[MeSH Terms] OR liver[All Fields] OR hepatic[All Fields] OR cirrhosis[All Fields] OR fibrosis[All Fields])

**C06. injection name literal without disease filter**

("salvianolate"[Title/Abstract] OR "salvianolic acids for injection"[Title/Abstract] OR "salvianolic acid for injection"[Title/Abstract] OR "depside salts"[Title/Abstract] OR (("Salvia miltiorrhiza"[Title/Abstract] OR Danshen[Title/Abstract]) AND polyphenol*[Title/Abstract] AND (inject*[Title/Abstract] OR intravenous[Title/Abstract])))

**C07. injection name literal liver**

("salvianolate"[Title/Abstract] OR "salvianolic acids for injection"[Title/Abstract] OR "salvianolic acid for injection"[Title/Abstract] OR "depside salts"[Title/Abstract] OR (("Salvia miltiorrhiza"[Title/Abstract] OR Danshen[Title/Abstract]) AND polyphenol*[Title/Abstract] AND (inject*[Title/Abstract] OR intravenous[Title/Abstract]))) AND ("Liver Diseases"[MeSH Terms] OR liver[All Fields] OR hepatic[All Fields] OR cirrhosis[All Fields] OR fibrosis[All Fields])

**PubMed retrieval data**

| Query | Returned records |
| --- | --- |
| Q1 | 257 |
| Q2 | 3 |
| C01 | 425 |
| C02 | 2513 |
| C03 | 71 |
| C04 | 2410 |
| C05 | 410 |
| C06 | 160 |
| C07 | 24 |
| Total raw returns | 6273 |

Q1 and Q2 shared one PMID, yielding 259 unique core/update records.

**S1.2. Web of Science Core Collection**

Query Builder was used with Editions set to All and no added date, language or document-type restriction. Coverage comprised all years available under institutional entitlement; individual sub-index entitlements and backfile starting years were not separately captured.

**WOS01. Danshen interventions and liver cellular evidence**

TS=("Salvia miltiorrhiza" OR Danshen OR "Dan Shen" OR salvianol* OR tanshinon* OR cryptotanshinon* OR dihydrotanshinon* OR "PF2401-SF" OR "PF2401 SF" OR "IH764-3" OR "IH764 3" OR "Fuzheng Huayu" OR "Fu Zheng Hua Yu" OR FZHY)
AND
TS=(hepatocyt* OR "hepatic stellate" OR "liver stellate" OR "hepatic fibrosis" OR "liver fibrosis" OR cirrhos* OR ((liver OR hepatic) AND fibros*))

**WOS02. Chemistry, formulation, pharmacokinetics and delivery**

TS=("Salvia miltiorrhiza" OR Danshen OR "Dan Shen" OR salvianol* OR tanshinon* OR cryptotanshinon* OR dihydrotanshinon* OR "PF2401-SF" OR "PF2401 SF" OR "IH764-3" OR "IH764 3")
AND
TS=(pharmacokinet* OR bioavailab* OR "drug metabolism" OR "metabolic stability" OR "protein binding" OR "plasma binding" OR "unbound concentration" OR "unbound fraction" OR "intracellular exposure" OR "cellular uptake" OR "cellular accumulation" OR "tissue distribution" OR "liver distribution" OR formulation* OR "drug delivery" OR nanoparticl* OR nanocapsul* OR nanocrystal* OR liposom* OR cyclodextrin* OR chromatograph* OR "mass spectrometry" OR HPLC OR UPLC OR "LC-MS" OR "LC-MS/MS" OR fingerprint* OR "chemical characterization" OR "chemical characterisation" OR "chemical composition" OR quantif* OR standardiz* OR standardis*)

**WOS03. Patient evidence and Danshen preparations**

TS=("Salvia miltiorrhiza" OR Danshen OR "Dan Shen" OR "Fuzheng Huayu" OR "Fu Zheng Hua Yu" OR FZHY OR salvianol* OR "depside salt" OR "depside salts" OR "salvianolic acid")
AND
TS=("liver fibrosis" OR "hepatic fibrosis" OR cirrhos* OR "chronic hepatitis" OR "hepatitis B" OR "hepatitis C" OR ((liver OR hepatic) AND fibros*))
AND
TS=(patient* OR clinical OR trial OR random* OR placebo OR cohort OR human* OR biopsy OR biopsies OR histolog*)

**Web of Science Core Collection retrieval data**

| Query | Exported rows |
| --- | --- |
| WOS01 | 335 |
| WOS02 | 2219 |
| WOS03 | 155 |
| Total raw exports | 2709 |

WOS02 contained one batch-boundary duplicate, leaving 2,218 records after within-query removal.

**S1.3. CAS SciFinder**

The following are the nine submitted inputs recorded in the saved guidance and author-confirmed execution. They are not separately captured platform-generated Query Interpretation screens. Chemistry and exposure searches did not require liver-fibrosis terms.

**SF01**

"PF2401-SF"

**SF02**

"PF2401 SF"

**SF03**

"Salvia miltiorrhiza" AND ("chemical composition" OR "chemical characterization" OR "chemical characterisation")

**SF04**

"Salvia miltiorrhiza" AND (fingerprint* OR "quantitative analysis" OR standardiz* OR standardis*)

**SF05**

"tanshinone IIA" AND (pharmacokinet* OR bioavailab* OR "protein binding" OR "tissue distribution" OR "cellular uptake" OR "intracellular concentration")

**SF06**

"salvianolic acid B" AND (pharmacokinet* OR bioavailab* OR "protein binding" OR "tissue distribution" OR "cellular uptake" OR "intracellular concentration")

**SF07**

cryptotanshinone AND (pharmacokinet* OR bioavailab* OR "protein binding" OR "tissue distribution" OR "cellular uptake" OR "intracellular concentration")

**SF08**

("dihydrotanshinone I" OR "15,16-dihydrotanshinone I") AND (pharmacokinet* OR bioavailab* OR "protein binding" OR "tissue distribution" OR "cellular uptake" OR "intracellular concentration")

**SF09**

("salvianolic acid B" OR "tanshinone IIA" OR cryptotanshinone OR "dihydrotanshinone I") AND (nanoparticle* OR nanocarrier* OR liposom* OR micelle* OR encapsulat* OR "drug delivery" OR "targeted delivery")

**CAS SciFinder retrieval data**

| Query | Exported rows |
| --- | --- |
| SF01 | 6 |
| SF02 | 6 |
| SF03 | 38 |
| SF04 | 668 |
| SF05 | 561 |
| SF06 | 500 |
| SF07 | 304 |
| SF08 | 125 |
| SF09 | 1,874 |
| Total raw exports | 4,082 |

**S1.4. Combined collection and selection data**

## Supplementary Table S1. All-database retrieval and selection flow

| Record or stage | Number | Interpretation |
| --- | --- | --- |
| Original PubMed raw returns | 260 | Archived 259 unique PMIDs |
| WoS raw exported records | 2,709 | Archived 335 + 2,219 + 155 |
| SciFinder raw exported records | 4,082 | Archived nine exported query sets |
| Patient-preparation PubMed raw returns | 6,013 | Archived seven queries; 2,578 within-query unique PMIDs |
| All database raw records | 13,064 | 21 queries before cross-query deduplication |
| Duplicate copies removed | 6,260 | Includes identity-reconciled duplicate copies |
| Deduplicated records | 6,804 | Raw records minus duplicate copies |
| Mixed-identity record removed | 1 | Removed before title/abstract screening |
| Title/abstract records screened | 6,803 | All database records together |
| Non-English records excluded before report review | 303 | Does not include the 24 language exclusions among reviewed reports |
| Other scope exclusions before report review | 5,685 | Record-level scope reasons preserved in the ledger |
| Database reports reviewed | 815 | Unified reviewed status |
| Database reports excluded after review | 658 | 24 language exclusions; one non-original report; 633 not selected after author appraisal |
| English database reports retained | 157 | Report-level eligibility; not 157 independent studies or citations |
| Candidate selection decisions pending | 0 | Author-confirmed review completed |
| Separately traced English report retained | 1 | Guo et al., 2026; outside the database denominator |
| All English reports retained | 158 | 157 database reports and one separately traced report |
| Requested full texts reported unobtainable | 0 | Author-confirmed acquisition of requested articles |
| Original PubMed records screened | 259 | 66 excluded before report review |
| Original PubMed reports reviewed | 193 | 95 not selected; 98 retained |

Table S1 summarizes the combined database flow and the original 259-PMID subgroup. The 157 retained English database reports and one separately traced report give 158 retained reports.

# Supplementary Table S2. Near-miss comparisons and additional source appraisal

| Study | Evidence retained | Unmet criterion / limitation | Source basis |
| --- | --- | --- | --- |
| PF2401-SF (Parajuli et al., 2013; Park et al., 2007) | Primary rat hepatocyte GCDC protection at 40 μg/mL; t-HSC/Cl-6 apoptosis at 20 μg/mL | Cross-study pairing; different concentration/time; no confirmed common experimental batch or paired cellular exposure | 2007 Methods 2.2–2.4, Figs 1–6; 2013 §§2.1–2.3,4.1,4.3 and Figs 1–4. The 20 μg/mL molar conversion is conditional, not measured. |
| Sal B (Lin et al., 2006b) | Same report: primary-rat-hepatocyte AST protection; primary-HSC activation suppression | 1-h hepatocyte endpoints vs 48-h HSC assays; suppression significant at 280 μM; the separate 1-h tolerance data do not show 48-h safety at that concentration | Methods 2.3–2.10, Tables 1–2 and Figs 3–7. Hepatocyte AST EC50 71.1 ± 7.2 μM. Conflicting MDA dose labels are not used to infer a new curve. |
| Sal A (Lin et al., 2006a) | 10 μM/24-h apoptosis in PDGF-stimulated HSC-T6; primary rat hepatocyte MTT tolerance at 0–20 μM/48 h | Same-study partial selectivity; no injured-hepatocyte rescue or paired exposure; unstimulated HSC-T6 is not an authenticated quiescent primary HSC | Methods for viability, TUNEL and cell cycle; apoptosis figures. PDGF-stimulated and unstimulated states kept separate. |
| Tanshinone IIA (Wu et al., 2019) | L02 injury-associated apoptosis reduced at 12.5 μM/24 h; LX-2 tested at 25 μM/24 h | L02 identity limitation; unequal nominal doses; no collagen-I/α-SMA decrease in LX-2 despite p-ERK change | Main Methods and Figs 3–5; supplied SI figures and analytical methods examined. Viability timings not assigned to all apoptosis assays. |
| FZHY serum (Wu et al., 2019; Tao et al., 2014) | L02 protection and LX-2 activation-marker suppression; a primary-hepatocyte DNA-transfer study supports an indirect route | Multibotanical serum cannot isolate Danshen; L02 limitation; state suppression without demonstrated persistence or HSC autonomy | 2019 main/SI; 2014 primary-hepatocyte and DNA-transfer methods. Drug carry-over must be controlled in a prospective autonomy test. |
| Cryptotanshinone (Hou et al., 2023; Li et al., 2024; Yin et al., 2009) | LX-2 apoptosis at 2.5–20 μM/24 h; separate 10 μM/48-h activation/metabolic assays | No matched injured-primary-hepatocyte arm; 40 μM L02 comparator has unverified separate timing; no verified washout/rechallenge | Source Methods/Fig 1; the 24-h viability duration is not automatically assigned to the L02 comparison. |
| DHI (Shen et al., 2025; Ge et al., 2017) | State suppression in LX-2/primary rat HSCs; ferroptosis evidence in primary mouse HSCs | Separate models and endpoints; no paired hepatocyte rescue; exact duration/rescue concentration of every mechanistic panel not verified | Ge main Fig 3 plus supplied supporting Fig S1; 24-h viability and 0.4/0.5 μM labels in ferroptosis report. Some supporting protocols unavailable. |
| Water extract (Oh et al., 2002) and related hepatoprotection (Lee et al., 2006) | BDL-rat hepatocyte apoptosis reduction; additional CCl4-associated hepatoprotection | In the BDL report α-SMA was unchanged; tissue improvement does not establish paired HSC fate or exposure | 2002 main article, water extract 200 mg/kg orally for 10 d; separate 2006 report remains cross-study context. |
| Tanshinone IIA globin nanoparticles (Meng et al., 2015) | Matched-dose IV parent-drug LC–MS/MS in plasma and whole liver; TAA-fibrosis results | Organ enrichment does not resolve cell-specific or unbound exposure; imaging signals analyzed separately | Meng 2015, Methods 2.9/2.11, Table 1, Figs 5–7 |
| Tanshinone IIA podocyte study (Zhu et al., 2024) | High-glucose MPC5 protection, ferroptosis-associated readouts and ACSL4 upregulation reversal | Renal model; neither liver dual-cell selectivity nor direct NQO1 mechanism | Zhu 2024, Methods and Results; 5/10/20 μM for 24 h |
| Danshensu in LX-2/T6 (Wang et al., 2023b) | 30 μM reduced collagen-I/CTGF; Figure 2 shows GPX4/SLC7A11 reduction; Figure 3 shows increased lipid ROS reversed by liproxstatin-1 | Headings/abstract/text disagree on direction; no viability/death-rescue chain. Lipid-ROS rescue alone does not establish HSC ferroptotic death. Methods SEM versus figure SD | Wang 2023, pages 2–5; inspected Figures 2–5 and full Methods/Results |

“Not verified” means that the specific condition was not independently confirmed from the available source. “Not reported” is used only for an inspected source.

# Supplementary Table S3. PF2401-SF composition and nominal conversions

Marker mass fractions are from the hepatocyte study (Park et al., 2007).

Molecular weights below are rounded PubChem values; calculations use the displayed values. Fixed main-table order is tanshinone I / tanshinone IIA / cryptotanshinone.

| Metabolite | Mass fraction | MW (g/mol) | 40 μg/mL: mass; μM | 20 μg/mL: mass; conditional μM |
| --- | --- | --- | --- | --- |
| Tanshinone I | 11.5% | 276.3 | 4.60 μg/mL; 16.6 | 2.30 μg/mL; 8.3 |
| Tanshinone IIA | 41.0% | 294.3 | 16.40 μg/mL; 55.7 | 8.20 μg/mL; 27.9 |
| Cryptotanshinone | 19.1% | 296.4 | 7.64 μg/mL; 25.8 | 3.82 μg/mL; 12.9 |

Nominal metabolite-equivalent concentration (μM) = fraction concentration (μg/mL) × mass fraction × 1,000 / molecular weight (g/mol).

| PK analyte (Park et al., 2008) | Reported total-plasma Cmax | Converted mean |
| --- | --- | --- |
| Tanshinone IIA | 2.78 ng/mL | 9.45 nM |
| Cryptotanshinone | 0.66 ng/mL | 2.23 nM |

Molar plasma concentration (nM) = concentration (ng/mL) × 1,000 / molecular weight (g/mol).

Molecular-weight sources: PubChem CIDs 114917, 164676 and 160254 (https://pubchem.ncbi.nlm.nih.gov/).

# Supplementary Table S4. Patient evidence by preparation and endpoint

| Preparation / reference | Design / administration | Reported outcome | Interpretive limitation |
| --- | --- | --- | --- |
| Oral Sal B (Liu et al., 2002) | 60 evaluable CHB patients; 6 months; interferon-γ comparison | Histological improvement 36.7% vs 30.0%; no significant between-group histological advantage | No paired cellular benefit/control measurements or achieved hepatocyte/HSC exposure. |
| FZHY + entecavir (Gui et al., 2020) | 52 randomized; 46 paired biopsies (22 vs 24) | ≥1-stage regression 18/22 vs 13/24; median Ishak reduction 1 in each group, not significant between groups | Multibotanical adjunct; binary response and stage-change endpoints differ; cannot isolate Danshen. |
| FZHY in CHC (Hassanein et al., 2022) | 118 randomized; 82 paired consensus-scored biopsies | F3/F4 comparison 57% vs 37%, P = 0.24; within-treatment stage comparisons are not treatment-versus-placebo efficacy | No significant cited subgroup treatment contrast; no paired cellular pharmacology. |
| Polyphenol injection surveillance (Cheng et al., 2020) | 2,633 exposed patients; 58 cases and 232 also-exposed matched controls | 58/2,633 (2.2%) abnormal liver-function events with Naranjo >5; 50 improved after withdrawal, eight after additional treatment | Main active constituent described as magnesium lithospermate B; no cohort-wide quantitative batch profile. No unexposed comparator; no attributable excess-risk or antifibrotic efficacy estimate. |
| Healthy-volunteer PK/tolerability (Jia et al., 2010; Cheng et al., 2023) | Depside salts in 12 volunteers; separate placebo-controlled Sal B phase 1 study | Preparation-specific exposure; phase 1 report included liver-test abnormalities despite no serious adverse events | Does not establish antifibrotic efficacy or long-term safety in liver-fibrosis patients. |
| Adjunctive FZHY pooled trial report (Guo et al., 2026) | Two open multicentre trials; 232 randomized, 218 analyzed; FZHY 1.6 g TID + entecavir for 96 weeks | Composite liver events 9/110 vs 21/108; adjusted HR 0.362 (0.159–0.828); individual bleeding/HCC/encephalopathy contrasts not significant | Post hoc; 14 post-randomization exclusions; limited events; no isolated Danshen effect or paired cellular exposure. Group by NCT02945982/NCT02945956 |

Clinical efficacy, safety surveillance and healthy-volunteer pharmacokinetics are separated in Table S4. The post hoc report (Guo et al., 2026) combines NCT02945982 and NCT02945956 and is not counted as two additional independent trials. Its unadjusted HR 0.408 (95% CI 0.187–0.892) and adjusted HR 0.362 (0.159–0.828) are distinct models. Ascites favoured adjunctive treatment; bleeding, HCC and encephalopathy contrasts were not significant. Routine HVPG measurement was not reported.

For injection safety (Cheng et al., 2020), the nine-study pooled event estimate, 2.17% (95% CI 1.05–3.58%), differs from the raw fraction 17/676 (2.51%) and from the cohort result 58/2,633 (2.2%). These estimates do not quantify attributable excess risk without an appropriate unexposed comparator. Registration chronology was not independently audited.

# Supplementary Table S5. Index of individual ConPhyMP assessments

| Reference | Preparation / material | Individual PDF filename |
| --- | --- | --- |
| Jia et al., 2010 | Danshen depside-salt preparation | Jia et al., 2010_ConPhyMP.pdf |
| Lee et al., 2006 | Aqueous Danshen extract | Lee et al., 2006_ConPhyMP.pdf |
| Oh et al., 2002 | Sm-X extract | Oh et al., 2002_ConPhyMP.pdf |
| Parajuli et al., 2015 | PF2401-SF | Parajuli et al., 2015_ConPhyMP.pdf |
| Parajuli et al., 2013 | PF2401-SF | Parajuli et al., 2013_ConPhyMP.pdf |
| Park et al., 2008 | PF2401-SF, PK report | Park et al., 2008_ConPhyMP.pdf |
| Park et al., 2007 | PF2401-SF | Park et al., 2007_ConPhyMP.pdf |
| Park et al., 2009 | PF2401-SF | Park et al., 2009_ConPhyMP.pdf |
| Tang et al., 2022 | Lipophilic Danshen extract | Tang et al., 2022_ConPhyMP.pdf |
| Wu et al., 2019 | FZHY / medicated serum | Wu et al., 2019_ConPhyMP.pdf |
| Yin et al., 2009 | Sm-SF fraction | Yin et al., 2009_ConPhyMP.pdf |
| Gui et al., 2020 | FZHY, HBV trial | Gui et al., 2020_ConPhyMP.pdf |
| Hassanein et al., 2022 | FZHY, HCV trial | Hassanein et al., 2022_ConPhyMP.pdf |
| Tao et al., 2014 | FZHY | Tao et al., 2014_ConPhyMP.pdf |
| Ping et al., 2024 | FZHY | Ping et al., 2024_ConPhyMP.pdf |
| Wang et al., 2020 | Liposoluble Danshen extract | Wang et al., 2020_ConPhyMP.pdf |
| Yang et al., 2015 | FZHY, PK report | Yang et al., 2015_ConPhyMP.pdf |
| Cheng et al., 2020 | Danshen polyphenol injection | Cheng et al., 2020_ConPhyMP.pdf |
| Guo et al., 2026 | FZHY, pooled HBV trial report | Guo et al., 2026_ConPhyMP.pdf |

Of the 74 cited references, 19 original reports using botanical materials, extracts, fractions or related preparations were selected for individual ConPhyMP assessment; 55 were outside this extract-checklist scope. The individual PDFs in Supplementary Data Sheet 1 contain the official ConPhyMP Tables 1 and 2a for the corresponding reports. Purified metabolites, chemically defined salts, synthetic derivatives and reconstructed mixtures of purified compounds are outside this scope; extract arms within mixed studies remain eligible. Applicability does not establish compliance. YES indicates explicit support, NO indicates incomplete or partial support or information not verified, and NA indicates genuine item-level inapplicability. No new botanical collection, extraction or analysis was performed for this review. The PDF filenames follow the format “Author et al., year_ConPhyMP.pdf”; the Reference column identifies the corresponding studies using the author–year citations in the main article.

# Supplementary Table S6. Prospective validation and methodological boundaries

| Dimension | Implementation / boundary |
| --- | --- |
| Intervention continuity | One characterized batch and common administration schedule; measure achieved exposure separately in the two compartments. Equal nominal treatment does not guarantee equal intracellular concentrations. |
| Hepatocyte benefit and safety | Separate injury-rescue and healthy-cell safety criteria with prespecified endpoints and acceptable uncertainty. Do not define safety using a high tolerated-toxicity EC90 alone. |
| HSC elimination / state control | Use concordant fate assays, morphology and rescue/perturbation where feasible. State suppression must be distinguished from apoptosis/ferroptosis and persistence after withdrawal. |
| State / subset selectivity | Assess fibrogenic markers with viability, lipid storage/quiescence programmes (e.g., LRAT, RBP1, PPARG) and functional hepatocyte support. Interpret RSPO3 with function rather than as a universal subset label (Sugimoto et al., 2025). |
| Heterogeneity and durability | Washout and renewed fibrogenic challenge; single-cell state resolution when mixed states obscure interpretation. Fate-transition claims need lineage or equivalent longitudinal evidence, not a single expression snapshot (Kisseleva et al., 2012). |
| Cell-autonomous HSC effects | Isolated HSC response, conditioned-medium transfer with residual-drug controls, and cell-specific perturbations. Consider injury reduction, macrophages and LSECs before attributing in vivo changes to direct HSC pharmacology (Tao et al., 2014; Ping et al., 2024). |
| Unbound cellular exposure | Measure total accumulation and cellular/medium binding; validate cell volume, carry-over, recovery, adsorption, metabolism and sequestration (Mateus et al., 2013; Mateus et al., 2017). Cu,cell = Ctotal,cell × fu,cell; Kp,uu,cell = Cu,cell / Cu,medium. Location of the target determines relevant exposure. |
| Target engagement | CETSA/TPP (Martinez Molina et al., 2013; Savitski et al., 2014) can be used without an engineered competitive tracer, subject to target detectability and orthogonal interpretation. BRET (Robers et al., 2015) requires a compatible tagged target/tracer. Negative thermal shifts do not exclude binding. |
| Hydrophilic vs lipophilic metabolites | For Sal B, verify uptake and target location. For lipophilic tanshinones, binding, adsorption and compartmental sequestration require particular care. These methods are proposals rather than validated universal assays for all candidates. |
| Quantitative window | Prespecify minimum hepatocyte benefit, safety and HSC-control thresholds; estimate donor-level concentration–effect relations and confidence intervals, including uncertainty in overlap. Report threshold sensitivity; do not calculate a window by dividing unmatched assay IC50 values. |
| Human systems | Injury-challenged primary human hepatocytes plus activated primary HSCs with healthy/state controls. Multicellular hiPSC liver models, liver-on-chip and human fibrotic precision-cut slices are complementary prospective systems; validate cell states, endpoints and washout suitability. |

## Four Pillars assessment for the present review

Pharmacological Requirements are addressed through traditional context, nominal molar concentrations, credible-model limitations, dose/exposure distinctions and separation of predictions from experimental evidence (Sections 2–5; Tables 1, S2, S3 and S6). Composition Requirements are addressed by botanical identity and source-specific processing/marker data, without transferring composition across unverified batches (Section 2; Tables S3 and S5; ConPhyMP forms). Basic Experimental and Ethical Requirements are addressed by the defined knowledge contribution, source ethics/provenance reporting and the absence of new author experiments (Sections 1 and 5; Table S5). Article-type Specific Requirements are addressed by the testable paired-cell question, justified critical narrative approach and critical appraisal of original-report validity (Introduction; Methods S1; Tables S1–S6). Source materials with missing information are not certified as compliant. Official source https://www.frontiersin.org/files/pdf/4_pillars_FULL_TEXT.pdf (consulted 8 October 2026).
